# Subacute thyroiditis following ginger (*Zingiber officinale*) consumption

**DOI:** 10.4103/0974-7788.59944

**Published:** 2010

**Authors:** Suzan Sanavi, Reza Afshar

**Affiliations:** *Shahed University, Faculty of Medicine, Mustafa Khomeini Hospital, Italia Street, Tehran, Iran*

**Keywords:** Ginger, subacute thyroiditis, *Zingiber officinale*

## Abstract

A woman with subacute thyroiditis following ginger consumption is presented. The diagnosis was confirmed by physical examination and laboratory tests, in two distinct episodes. The patient was cured and recommended to refuse ginger consumption.

## INTRODUCTION

Subacute thyroiditis is presumed to be caused by a viral infection or a postviral inflammatory process, and autoimmunity may play a secondary role in this disorder.[1] The resulting inflammation damages thyroid follicles and large amounts of thyroid hormones are released into the circulation, resulting in clinical and biochemical thyrotoxicosis. This state is transient, because new hormone synthesis ceases due to follicular cells damage and inhibition of thyrotropin (TSH) secretion by the increased thyroid hormone concentrations.[2,3] The thyroid gland is usually slightly or moderately diffusely enlarged, and nearly always tender. Thyrotoxicosis usually subsides in 2–6 weeks, even if the patient is not treated.[4,5] Subacute thyroiditis is fundamentally a clinical diagnosis and a high erythrocyte sedimentation rate (ESR) is strong confirmatory evidence. Ginger has been used as a heat producer among Iranian population and some studies have shown its effects on metabolism. Thyroid hormones have a regulatory role on metabolism which may be influenced by ginger. To our knowledge this case is a first case that indicates the effects of ginger on thyroid function.

## CASE REPORT

**Figure 1:**
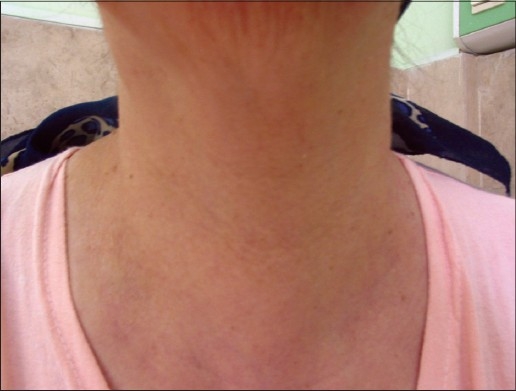
Anterior view of neck.

A 34-year-old female was referred to a private office, because of sudden onset of severe pain at the back of the mouth and neck radiating to jaw, difficulty in swallowing, hoarse voice, mild fever and palpitation. On physical examination, a red throat without any exudates was observed but cervical lymph nodes enlargement and coryza signs were absent. The thyroid gland was diffusely enlarged (grade Ib or only visible in neck extension) and severely tender [Figure 1]. Patient's temperature was 38°C and heart rate (HR = 120/min) had disproportionately increased. There was no history of prior goiter, abnormal thyroid function, allergic reactions and recent viral infection. She was not in postpartum period. An interesting finding in her history was ginger powder consumption (1 teaspoonful) with honey (1 tablespoonful) for 10 nights.The thyroid function tests (TFT) revealed thyrotoxicosis. The new TFT indicated the same results. Table 1 shows useful diagnostic laboratory findings:

**Table 1:** Patient diagnostic laboratory findings

Laboratory data	1^st^ time	2^nd^ time(1 year later)
T4 (mcg/ml)	20	15
T3 (ng/ml)	320	260
TSH (mU/L)	<0.01 (IRMA)	<0.01 (IRMA)
ESR (mm/h)	70	50
WBC (mm3)	14000 (PMN 35%)	12000
Anti-thyroglobulin (IU/ml)	Not detectable	Not detectable

Normal ranges: T4:4.4–11,T3:60–181,TSH:0.5–5

Radioactive iodine uptake of thyroid gland was diminished, compatible with the diagnosis of subacute thyroiditis. She had been treated with nonsteroidal antiinflammatory drugs (NSAIDs), propranolol and prednisolone by her physician. Although, she did not consume prescribed drugs regularly, she was relieved after 5 months. One year later, following ginger candy consumption (30 g), she experienced the same previous symptoms in milder form, 24 h after ginger intake. She was referred to us for evaluation, and with the same diagnosis, TFT was requested that revealed thyrotoxicosis [Table 1]. Fortunately, thyrotoxicosis subsided within four weeks only with beta-blocker therapy and patient refused to consume prescribed antiinflammatory drugs, again.

## DISCUSSION

Ginger is commonly used as a cooking spice throughout the world. It is the rhizome of the perennial plant *Zingiber officinale* in the family Zingiberaceae. The ginger plant has a long history of cultivation, known to have originated in Asia and then spread to other parts of the world.[6] Ginger root is available in various forms including: dried root and powdered ginger. The primary known constitutes of ginger root are gingerol, zingibain, bisabolene, oleoresins, starch, essential oil, mucilage and protein.[7] Ginger contains up to three percent of an essential oil that gives it fragrance. The main constituents are sesquiterpenoids with zingiberene as the main component. Lesser amounts of other sesquiterpenoids (beta- sesquiphellandrene, bisabolene and farnesene) and a small monoterpenoid fraction (beta-phellandrene, cineol and citral) have also been identified. In addition, it is a minor chemical irritant and has sialagogue action, stimulating the production of saliva, which makes it easier to swallow.[8] The medical form of ginger historically was called ‘Jamaica ginger’ and has been used to treat nausea related to both motion sickness and morning sickness,[7,9] asthma and bronchitis by loosening and expelling mucus from the lungs, dyspepsia as a carminative, thrombosis as a thromboxane synthetase inhibitor,[7] pain and inflammation due to arthritis, rheumatism and muscle spasm as an antiinflammatory agent[10] and to break fevers by increasing perspiration.[11] The ginger has antioxidative, antidiabetic effects and pressor activity in rats.[12,13,7] It is a safe herb and up to now, significant side effects such as thyroiditis have not been reported. Despite its experimental antioxidative and inhibitory effects on metabolic rate, which theoretically may lead to decreased thyroid hormone synthesis, in this report, ginger is presumed to play a role in subacute thyroiditis induction. Inhibitory effect of ginger on metabolic rate and adenylate energy status[14] may damage the integrity of membranes surrounding the thyroid hormones in follicles and eventually release hormones into circulation. Also, subacute thyroiditis may be induced due to an autoimmune process by ginger as an antigen, which changes antigenic properties of thyroid follicular cells. Although, allergic reactions to ginger are generally reported as skin rash, it may promote wider inflammatory responses. It would be interesting to investigate whether this alleged adverse effect has any genetic predisposition. Further research is needed to understand the possible effects of ginger on thyroid gland.
